# Non-contiguous finished genome sequence of *Ornithobacterium rhinotracheale* strain H06-030791

**DOI:** 10.1186/1944-3277-9-14

**Published:** 2014-12-08

**Authors:** Emilie S Zehr, Darrell O Bayles, William D Boatwright, Louisa B Tabatabai, Karen B Register

**Affiliations:** 1 Ruminant Diseases and Immunology Research Unit, U. S. Department of Agriculture, Agricultural Research Service, National Animal Disease Center, Ames, IA, USA; 2 Infectious Bacterial Diseases Research Unit, U. S. Department of Agriculture, Agricultural Research Service, National Animal Disease Center, Ames, IA, USA; 3 Roy J. Carver Department of Biochemistry, Biophysics and Molecular Biology, Iowa State University, Ames, IA, USA

**Keywords:** *Ornithobacterium rhinotracheale*, Respiratory disease, Poultry, Genome sequence

## Abstract

The Gram-negative, pleomorphic, rod-shaped bacterium *Ornithobacterium rhinotracheale* is a cause of pneumonia and airsacculitis in poultry. It is a member of the family *Flavobacteriaceae* of the phylum “*Bacteroidetes”. O. rhinotracheale* strain H06-030791 was isolated from the lung of a turkey in North Carolina in 2006. Its genome consists of a circular chromosome of 2,319,034 bp in length with a total of 2243 protein-coding genes and nine RNA genes. Genome sequences are available for two additional strains of *O. rhinotracheale*, isolated in 1988 and 1995, the latter described in a companion genome report in this issue of SIGS. The genome sequence of *O. rhinotracheale* strain H06-030791, a more contemporary isolate, will be of value in establishing core and pan-genomes for *O. rhinotracheale* and elucidating its evolutionary history.

## Introduction

*Ornithobacterium rhinotracheale* has been implicated as a cause of respiratory disease in domesticated fowl since at least 1981 [1]. Initially characterized as a phenotypically unusual bacterium of uncertain identity [2], Vandamme et al. [3] further characterized and named *O. rhinotracheale* in 1994. *O. rhinotracheale* is a global pathogen in farmed turkeys and chickens as well as a variety of other domesticated and wild birds, including chukar partridges, geese, ducks, guinea fowl, ostriches, gulls, pheasants, partridges, pigeons, quail, rooks, and falcons [4,5]. Based on the reactivity of heat-extracted antigens with monospecific antisera, 18 serotypes of *O. rhinotracheale* have been defined, designated as A through R [1,4], although not all isolates are typeable. The most common clinical signs of disease related to *O. rhinotracheale* are tracheitis, pneumonia, airsacculitis, sinusitis, and pericarditis [1,4]. The bacterium is responsible for substantial economic losses to the poultry industry worldwide, resulting from decreased egg production, reduced eggshell quality and hatchability, reduced weight gain, increased mortality, and increased condemnation rates [6-9]. Whole-cell bacterin and live, attenuated vaccines have met with variable success, likely due to the lack of cross-protection against heterologous serotypes. Recent studies have identified antigens that appear to provide cross-protective immunity when formulated as a recombinant, multi-component subunit vaccine [10].

*O. rhinotracheale* strain H06-030791 was isolated in 2006 from the lung of a turkey in North Carolina and subsequently determined to be serotype A in the laboratory of Dr. K. V. Nagaraja at the University of Minnesota, St. Paul, MN. Further study revealed that growth of *O. rhinotracheale* strain H06-030791 *in vitro* is unaffected by the presence of an iron chelator [11] a phenotype not shared by most of the other field isolates tested. Whether or how this attribute plays a role in disease is not yet clear. Although *O. rhinotracheale* has generally been considered nonhemolytic on blood agar, Tabatabai et al. [12] documented strong β-hemolytic activity of *O. rhinotracheale* strain H06-030791 and suggested that a hemolysin-like protein may function as a virulence factor. Here we present a description of the non-contiguous finished genome of *O. rhinotracheale* strain H06-030791 and its annotation. This isolate (alias P5932) was provided to the National Animal Disease Center by the University of Minnesota and is available from the National Animal Disease Center Biological Agent Archive.

## Organism information

### Classification and features

**Figure 1:**
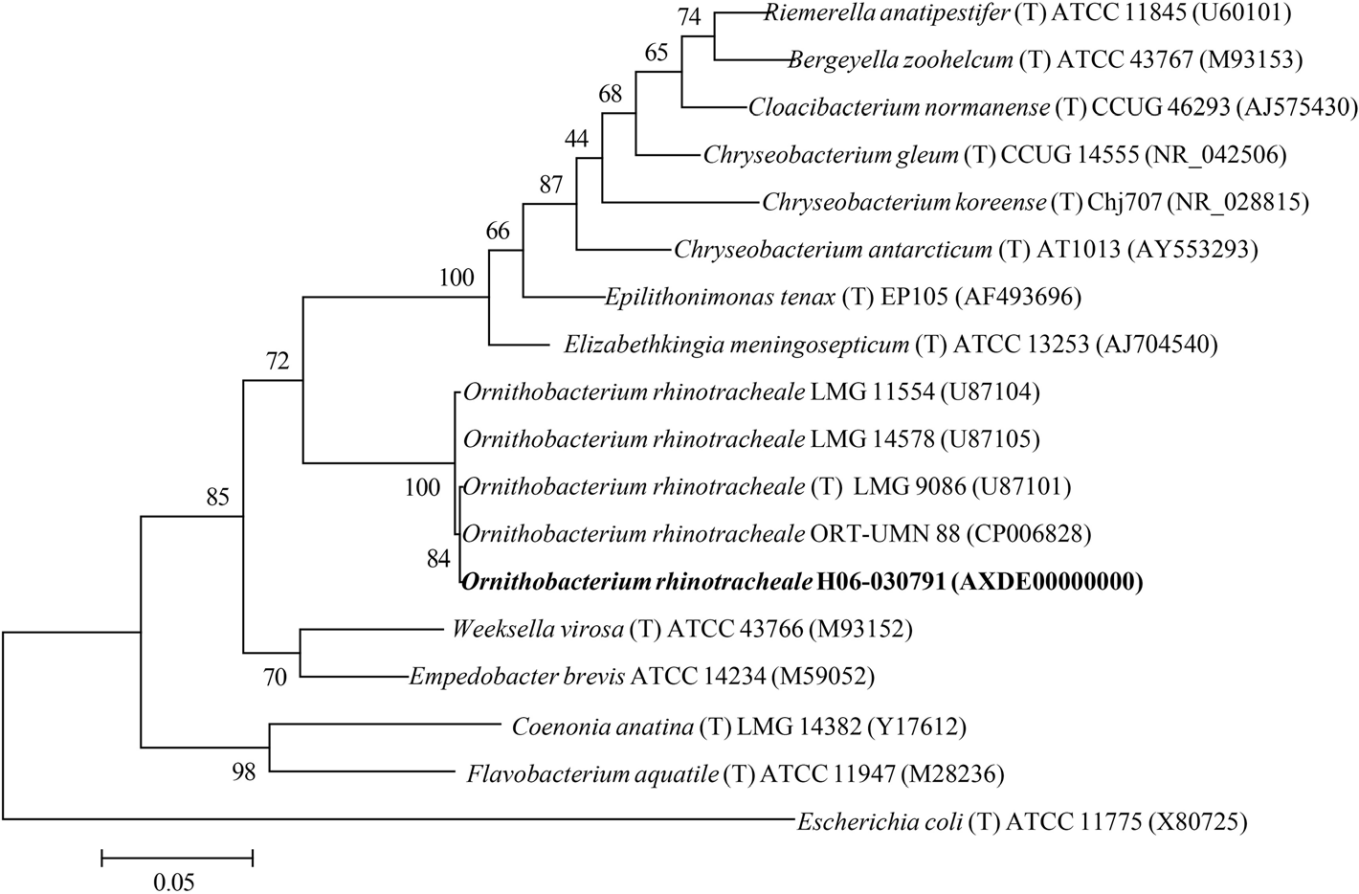
**Phylogenetic tree based on 16S rRNA showing the position of *O. rhinotracheale *strain H06-030791 (highlighted in bold) in relation to other *O. rhinotracheale *isolates for which sequence is available and to the type strains (T) of closely related species and genera within the family *Flavobacteriaceae*. ***Escherichia coli* (a member of the *Enterobacteriaceae* family) was included as an outgroup. An internal region of the 16S RNA gene (1251 bp with no gap-containing sites) was aligned using CLUSTALW and phylogenetic inferences were obtained using the maximum likelihood method and the Jukes-Cantor model within MEGA version 5.10 software [37]. Numbers at the nodes are percentages of bootstrap values obtained by repeating the analysis 1000 times to generate a majority consensus tree. GenBank accession numbers for the DNA sequences used are shown in parentheses. The scale bar represents 5% substitution per nucleotide position.

The genus *Ornithobacterium* belongs to the class *Flavobacteriia* and is in the family *Flavobacteriaceae*[13] (Table 1). *O. rhinotracheale* is the sole species within the genus. Phylogenetic analysis based on 16S ribosomal RNA of *O. rhinotracheale* and other genera within the *Flavobacteriaceae* family is shown in Figure 1. The 16S rRNA sequences of *O. rhinotracheale* strain H06-030791 and the type strain, LMG 9086, share 99.9% nucleotide sequence identity. Three rRNA loci were found in the genome of *O. rhinotracheale* strain H06-030791. All *O. rhinotracheale* strains in Figure 1 were isolated from turkeys, with the exception of strain LMG 11554, which was cultured from a rook.

**Figure 2:**
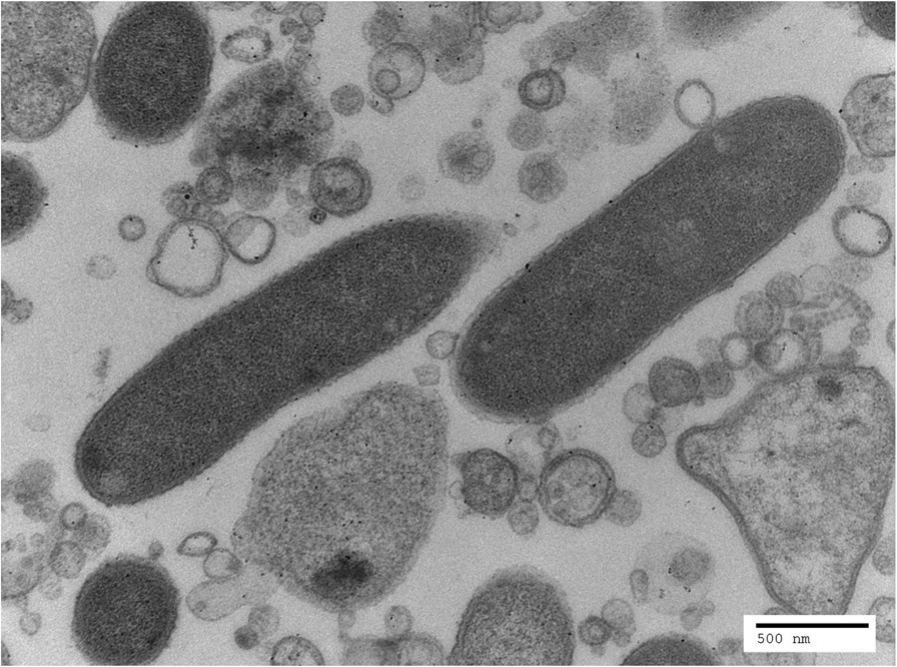
**Transmission electron micrograph of *O. rhinotracheale *strain H06-030791 cells cultured in broth, using a Tecnai G**^**2 **^**(FEI, Hillsboro, OR) at an operating voltage of 80 kV.** The average length of representative cells was 1.93 μm and the average width was 0.48 μm. The scale bar represents 500 nm.

*O. rhinotracheale* strain H06-030791 is a Gram-negative, pleomorphic rod, when grown in broth medium, ranging from 1.57-2.19 μm (mean, 1.93 μm) in length and 0.42-0.64 μm (mean, 0..48 μm) in width (Figure 2). The bacterium is nonmotile and microaerophilic, and prefers a 7.5% CO_2_ humidified atmosphere from 30°C to 42°C for growth. Colonies are approximately 1 mm in diameter and yellowish in color after 48 h incubation at 37°C on blood agar. Although *O. rhinotracheale* type strain LMG 9086 is nonhemolytic [3], *O. rhinotracheale* strain H06-030791 is β-hemolytic on 5% sheep blood agar [12].

Biochemical tests for *O. rhinotracheale* strains can yield variable results [1]. After seven days of incubation at 37°C, *O. rhinotracheale* strain H06-030791 is weakly acidic on a triple sugar iron agar slant and does not produce hydrogen sulfide or gas. Dextrose is weakly fermented with or without the addition of 2% chicken serum, while galactose and lactose are weakly fermented only with the addition of 2% chicken serum. Sucrose, sorbitol, xylose, and mannitol are not fermented with or without the addition of 2% chicken serum. The isolate is lysine decarboxylase positive, ornithine decarboxylase negative, and urease negative.

**Table 1:** **Classification and general features of *O. rhinotracheale *** **strain H06-030791 in accordance with the MIGS recommendations**[14]

**MIGS ID**	**Property**	**Term**	**Evidence code**^**a**^
	Current classification	Domain “*Bacteria”*	TAS [15,16]
		Phylum “*Bacteroidetes”*	TAS [17,18]
		Class “*Flavobacteriia”*	TAS [19,20]
		Order *Flavobacteriales*	TAS [21,22]
		Family *Flavobacteriaceae*	TAS [23-25]
		Genus *Ornithobacterium*	TAS [26,27]
		Species *rhinotracheale*	TAS [26,27]
MIGS-7	Subspecific genetic lineage (strain)	Strain H06-030791	TAS [11]
		Serotype A	IDA
	Gram stain	Negative	TAS [1,4]
	Cell shape	Pleomorphic rod	TAS [1,4]
	Motility	Nonmotile	TAS [1,4]
	Sporulation	Non-sporulating	TAS [1,4]
	Temperature range	Mesophile (30°C-42°C)	TAS [1,4]
	Optimum temperature	37°C	TAS [1,4]
MIGS-6.2	pH range; Optimum	7.2-7.6 (BHI); 7.4	TAS [1], IDA
	Carbon source	Saccharolytic (glucose)	TAS [4]
MIGS-6	Habitat	Respiratory tract of birds worldwide	TAS [1,4]
MIGS-6.3	Salinity	Growth in BHI broth, (0.75% salts)	TAS [1], IDA
MIGS-22	Oxygen requirement	Microaerophilic, anaerobic, or aerobic	TAS [1,4]
	Energy metabolism	Chemoorganotroph	TAS [4]
MIGS-15	Biotic relationship	Parasitic	TAS [4]
MIGS-14	Pathogenicity	Pneumonia, airsacculitis, tracheitis, pericarditis	TAS [1,4]
MIGS-16	Specific host	Poultry	TAS [1,4]
MIGS-18	Health status of host	Symptomatic	TAS [11]
	Biosafety level	2 t	TAS [28]
MIGS-19	Trophic level	Chemoheterotroph	TAS [4]
MIGS-23.1	Isolation	Turkey lung	TAS [11]
MIGS-4	Geographic location	North Carolina, USA	TAS [11]
MIGS-5	Time of sample collection	2006	NAS
MIGS-4.1	Latitude	Not reported	
MIGS-4.2	Longitude	Not reported	
MIGS-4.3	Depth	Not reported	
MIGS-4.4	Altitude	Not reported	

^a^Evidence codes - IDA: Inferred from Direct Assay by Dr. K. V. Nagaraja, University of Minnesota, St. Paul, MN; TAS: Traceable Author Statement (i.e., a direct report exists in the literature); NAS: Non-traceable Author Statement (i.e., not directly observed for the living, isolated sample, but based on a generally accepted property for the species, or anecdotal evidence). Evidence codes are from the Gene Ontology project [29].

## Genome sequencing and annotation

### Genome project history

Genome sequences are currently available for only two additional strains of *O. rhinotracheale*, the type strain LMG 9086 (isolated in 1988) and strain ORT-UMN 88 (isolated in 1995; see companion report in this issue of SIGS). *O. rhinotracheale* strain H06-030791 was selected for sequencing to provide a basis for comparative analysis of contemporary versus historical isolates. Additionally, *O. rhinotracheale* strain H06-030791 possesses phenotypic traits unique from those of *O. rhinotracheale* strain LMG 9086 and *O. rhinotracheale* strain ORT-UMN 88 [11,12] that may permit a more accurate representation of the core and pan-genomes of *O. rhinotracheale*. The Whole Genome Shotgun project and non-contiguous finished genome sequence of *O. rhinotracheale* strain H06-030791 has been deposited in DDBJ/EMBL/GenBank under accession no. AXDE00000000. Sequencing, finishing, and final annotation were performed at the DNA Facility of Iowa State University and the National Animal Disease Center, Ames IA. A summary of the project information is given in Table 2.

**Table 2:** **Project information of *O. rhinotracheale *** **strain H06-030791**

**MIGS ID**	**Property**	**Term**
MIGS-31	Finishing quality	Non-contiguous finished
MIGS-28	Libraries used	Three genomic libraries: two shotgun libraries, one mate-pair library (8 kb insert size)
MIGS-29	Sequencing platforms	Illumina GA II, Roche GS FLX Titanium, Sanger
MIGS-31.2	Fold coverage	48x (26× Roche FLX, 23× Illumina); final SEQuel error correction with 100× Illumina
MIGS-30	Assemblers	MIRA v3.4.0, Roche gsAssembler v2.8
MIGS-32	Gene calling method	GeneMarkS + (NCBI PGAP)
	GenBank ID	AXDE00000000
	GenBank Date of Release	September 22, 2014
	GOLD ID	Gi0071045
	NCBI project ID	219465
	Project relevance	Poultry respiratory pathogen
MIGS-13	Source material identifier	H06-030791

### Growth conditions and DNA isolation

A clonal population of *O. rhinotracheale* strain H06-030791 was derived from a single colony serially passaged three times and archived at −80°C for future analysis. The bacterium was grown on 5% sheep blood agar plates (Becton, Dickinson and Company, Sparks, MD) incubated for 48 h at 37°C with 7.5% CO_2_ and 15% humidity. Colonies were used to inoculate 5 ml of brain heart infusion broth in a snap-cap tube which was incubated at 37°C for 24 h with rotation at 100 rpm. Twenty ml of these BHI cultures were inoculated into 100 ml of fresh BHI in a 250-ml flask and incubated at 37°C for 48 h with rotation at 75 rpm (final OD_600 =_ 0.278). An aliquot was plated on 5% sheep blood agar to confirm purity and 20 ml was removed for DNA preparation. Cells were pelleted successively into one 2-ml centrifuge tube at 16,000 × g. Genomic DNA was isolated using the Wizard Genomic DNA Purification Kit (Promega Corporation, Madison, WI) with the following modifications: the cell pellet was resuspended in 480 μl of 200 mM EDTA, 60 μl of 10 mg/ml lysozyme, and 60 μl of double distilled water prior to lysis, then 10 μl of 10 mg/ml RNase solution was added to the cell lysate. The precipitated genomic DNA was rehydrated at 65°C for 1 h in 10 mM Tris–HCl, pH 8.5, evaluated on a 6% agarose gel to verify the lack of low molecular weight fragments, and quantified using the Quant-iT PicoGreen ds DNA Assay Kit (Invitrogen, Carlsbad, CA).

### Genome sequencing and assembly

A scaffolded genome was assembled using MIRA v. 3.4 [30] and the Roche gsAssembler v. 2.6 to achieve 49 × total genome coverage through the assembly of Roche GS FLX shotgun, GS FLX large insert (8.3 kb) mate pair, Illumina 75-bp single direction, and Illumina 2 × 75 bp paired-end sequencing reads. Some of remaining sequencing gaps in the scaffolded assembly were PCR amplified and sequenced by the Sanger method. GAP5 [31], from the Staden Package, was used as the editor for incorporating the gap-closing sequences, ultimately resulting in a high quality assembly consisting of eight contigs and seven gaps. (The genome start and end points are in a complete contig that was intentionally split to facilitate comparisons to a completed genome of the same genus and species.) Base calling errors in the genome assembly were corrected by using SEQuel [32] to map Illumina reads back to the contigs at approximately 100 × total coverage.

### Genome annotation

The assembled genome was submitted to the National Center for Biotechnology Information (Bethesda, MD) through the Whole Genome Shotgun genome sequencing portal [33] and annotated with the NCBI Prokaryotic Genome Annotation Pipeline. Signal peptides were distinguished from transmembrane regions by using SignalP 4.0 software [34], transmembrane helices were predicted with the method of Krogh et al. [35], and the CRISPR motif was discovered with a web tool described by Griss et al. [36].

### Genome properties

**Figure 3:**
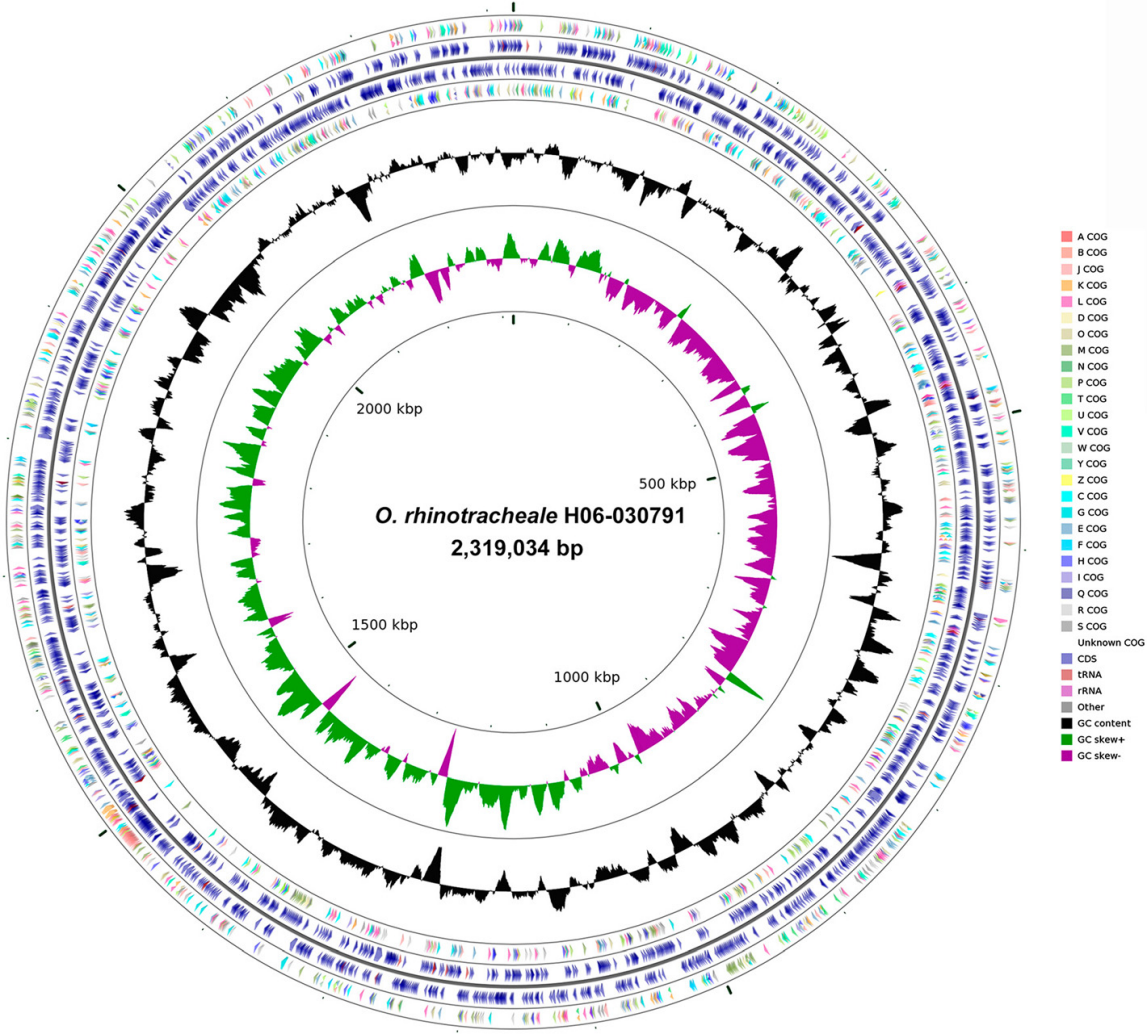
**Graphical map of the *O. rhinotracheale *strain H06-030791 chromosome.** From outside to the center: genes on forward strand (color by COG categories), CDS on forward strand, tRNA, rRNA, other; CDS on reverse strand, tRNA, rRNA, other, genes on reverse strand (color by COG categories); GC content; GC skew, where green indicates positive values and magenta indicates negative values.

The genome properties and statistics of *O. rhinotracheale* strain H06-030791 (Accession AXDE00000000) are presented in Tables 3 and 4 and Figure 3. The non-contiguous finished genome consists of a circular 2,319,034 bp chromosome with a 34.53% G + C content and no plasmids. Of the 2,300 genes predicted, 2,243 are protein-coding genes, six are pseudogenes, and nine are RNA genes. The percentage of the protein-coding genes that were assigned a putative function is 47.17%. The distribution of genes into COGs functional categories is presented in Table 4. One CRISPR motif was also detected.

**Table 3:** **Genome statistics of *O. rhinotracheale *** **strain H06-030791**

**Attribute**	**Genome (total)**	
	**Value**	**% of total**^**a**^
Genome size (bp)	2,319,034	100.00%
DNA coding (bp)	2,100,363	90.57%
DNA G + C (bp)	800,726	34.53%
Total genes	2300	100.00%
Protein-coding genes	2243	97.52%
RNA genes	9	0.39%
rRNA operons	3	
tRNA genes	42	1.83%
Pseudo genes	6	0.27%
Genes with function prediction	1058	47.17%
Genes assigned to COGs	1384	61.70%
Genes assigned Pfam domains	1487	66.30%
Genes with signal peptides	254	11.32%
Genes with transmembrane helices	471	21.00%
CRISPR repeats	1	

^a^The total is based on either the size of the genome in base pairs or the total number of protein coding genes in the annotated genome.

**Table 4:** **Number of genes associated with the 25 general COG functional categories of *O. rhinotracheale *** **strain H06-030791**

**Code**	**Value**	**% age**^**a**^	**Description**
J	133	5.7	Translation, ribosomal structure and biogenesis
A	0	0	RNA processing and modification
K	47	2.01	Transcription
L	118	5.06	Replication, recombination and repair
B	0	0	Chromatin structure and dynamics
D	19	0.81	Cell cycle control, cell division, chromosome partitioning
Y	0	0	Nuclear structure
V	36	1.54	Defense mechanisms
T	24	1.03	Signal transduction mechanisms
M	122	5.23	Cell wall/membrane biogenesis
N	3	0.13	Cell motility
Z	0	0	Cytoskeleton
W	0	0	Extracellular structures
U	27	1.16	Intracellular trafficking and secretion, and vesicular transport
O	66	2.83	Posttranslational modification, protein turnover, chaperones
C	76	3.26	Energy production and conversion
G	78	3.34	Carbohydrate transport and metabolism
E	112	4.8	Amino acid transport and metabolism
F	52	2.23	Nucleotide transport and metabolism
H	91	3.9	Coenzyme transport and metabolism
I	42	1.8	Lipid transport and metabolism
P	82	3.51	Inorganic ion transport and metabolism
Q	17	0.73	Secondary metabolites biosynthesis, transport and catabolism
R	150	6.43	General function prediction only
S	89	3.81	Function unknown
-	949	40.48	Not in COGs

^a^The total is based on the total number of protein coding genes in the annotated genome.

## Conclusions

Prior to this report only a single genome sequence was available for *O. rhinotracheale*, from the type strain LMG 9086, and no corresponding analysis of an *O. rhinotracheale* genome has been published. Examination of the aligned genomes of these isolates revealed that rearrangements and inversions are the major distinguishing features. Relative to LMG 9086, the genome of H06-030791 contains a single rearrangement of ~31 Kb, a single inversion of ~17 Kb and three regions that are both inverted and rearranged, varying from ~59-354 Kb each, many with a transposase or transposon present at one terminus. Thus, mobile elements may play a role in shaping genome structure and evolution of *O. rhinotracheale*. Within one of the inverted and rearranged segments of H06-030791 is an apparent deletion of ~37 Kb found in LMG 9086, comprised primarily of CDSs annotated as hypothetical proteins but also including a holin family protein, an ATP-dependent serine protease, a helix-turn-helix protein and several phage-related proteins. Owing to gaps in the H06-030791 genome, the putative deletion requires confirmation but it does lie well within the boundaries of the contig in which it is found and adjacent sequences are syntenous with the LMG 9086 genome. Also within the same rearranged/inverted region is an insertion in H06-030791 with five predicted CDSs, four annotated as hypothetical proteins and one as a multidrug ABC transporter.

Notable phenotypes associated with H06-030791 but not the type strain include β-hemolytic activity [12] and the ability to grow in the presence of an iron chelator [11]. Only three CDSs whose annotations suggest a function in hemolytic activity were apparent in H06-030791. Identical or nearly identical homologs were found in the LMG 9086 genome. One additional CDS annotated in LMG 9086 as a hemolysin was also found in H06-030791, identical in sequence but annotated there as a glycerol acyltransferase. Among 15 CDSs collectively found in H06-030791 and LMG 9086 whose annotations suggest a role in iron acquisition or transport, only one was found to have considerable sequence divergence. The integral membrane protein and ferrous iron transporter FeoB is predicted to be identical in both isolates over the N-terminal 395 amino acids but only 94.7% identical over the C-terminal 301 amino acids. Motifs found within the divergent region of the protein include a ferrous iron transport protein B C terminus (PF07664.7) flanked by two gate nucleoside recognition domains (PF07670.9). As these are believed to comprise the membrane pore region, sequence heterogeneity may perhaps affect the specificity of transport. Other homologs in H06-030791 and LMG 9086 with obvious sequence divergence include several annotated as hypothetical proteins, a transcriptional regulator/sugar kinase with a highly divergent stretch of ~50 bp, a Crp/Fnr family transcriptional regulator with nearly all amino acid substitutions in the cyclic nucleotide binding domain (PF00027.24) of the predicted protein and a PAO141 family polyphosphate kinase 2, with substitutions concentrated in the polyphosphate kinase 2 domain (PF03976.9).

The genome sequence of H06-030791, together with those of the type strain and an additional, recently sequenced isolate [38] will provide a framework for future investigations designed to elucidate the genetic basis of virulence in *O. rhinotracheale* and for understanding genome structure and evolution.

## Competing interests

The authors declare that they have no competing interests.

## Authors’ contributions

EZ participated in genome sequencing and drafted the original manuscript. DB directed genome sequence assembly and bioinformatics analyses. WB participated in genome sequencing and post-sequencing analyses. LT conceived of the study and participated in genome sequencing. KR participated in post-sequencing analysis and revised the manuscript. All authors read and approved the final manuscript.

## References

[B1] Chin RP Van Empel PCM Hafez HM Saif YM *Ornithobacterium rhinotracheale* infection Diseases of Poultry 2008 12 Ames, IA: Blackwell Publishing 765 74

[B2] Charlton BR Channing-Santiago SE Bickford AA Cardona CJ Chin RP Cooper GL Droual R Jeffrey JS Meteyer CU Shivaprasad HL Walker RL Preliminary characterization of a pleomorphic gram-negative rod associated with avian respiratory disease J Vet Diagn Invest 1993 5 47 51 PMID: 8466980 10.1177/104063879300500111 8466980

[B3] Vandamme P Segers P Vancanneyt M Van Hove K Mutters R Hommez J Dewhirst F Paster B Kersters K Falsen E Devriese LA Bisgaard M Hinz K-H Mannheim W *Ornithobacterium rhinotracheale* gen. nov. sp. nov., isolated from the avian respiratory tract Int J System Bacteriol 1994 44 1 24 37 PMID: 8123560 10.1099/00207713-44-1-24 8123560

[B4] Hafez HM Vandamme P Krieg NR, Staley JT, Brown DR, Hedlund BP, Paster BJ, Ward NL, Ludwig W, Whitman WB Genus XXXIX. *Ornithobacterium* Vandamme, Segers, Vancanneyt, Van Hove, Mutters, Hommez, Dewhirst, Paster, Kersters, Falsen, Devriese, Bisgaard, Hinz and Mannheim 1994b, 35^VP^ Bergey’s Manual of Systematic Bacteriology, Volume 4 2011 2 New York: Springer 250 4

[B5] Hafez HM Lierz M *Ornithobacterium rhinotracheale* in nestling falcons Avian Dis 2010 54 1 161 3 PMID: 20408418 10.1637/9008-080309-Case.1 20408418

[B6] van Veen L Gruys E Frik K van Empel P Increased condemnation of broilers associated with *Ornithobacterium rhinotracheale* Vet Rec 2000 147 15 422 3 doi:10.1136/vr.147.15.422 10.1136/vr.147.15.422 11072989

[B7] Sprenger SJ Halvorson DA Nagaraja KV Spasojevic R Dutton RS Shaw DP *Ornithobacterium rhinotracheale* infection in commercial laying-type chickens Avian Dis 2000 44 3 725 9 PMID: 11007028 10.2307/1593120 11007028

[B8] De Rosa M Droual R Chin RP Shivaprasad HL Walker RL *Ornithobacterium rhinotracheale* infection in turkey breeders Avian Dis 1996 40 4 865 74 doi:10.2307/1592311 10.2307/1592311 8980819

[B9] van Empel PCM Hafez HM *Ornithobacterium rhinotracheale*: a review Avian Pathol 1999 28 3 217 27 doi:10.1080/03079459994704 10.1080/03079459994704 26915377

[B10] Schuijffel DF van Empel PCM Segers RPAM Van Putten JPM Nuijten PJM Vaccine potential of recombinant *Ornithobacterium rhinotracheale* antigens Vaccine 2006 24 1858 67 doi:10.1016/j.vaccine.2005.10.031 10.1016/j.vaccine.2005.10.031 16318896

[B11] Tabatabai LB Zehr ES Zimmerli MK Nagaraja KV Iron acquisition by Ornithobacterium rhinotracheale Avian Dis 2008 52 3 419 25 PMID: 18939629 10.1637/8185-113007-Reg 18939629

[B12] Tabatabai LB Zimmerli MK Zehr ES Briggs RE Tatum FM *Ornithobacterium rhinotracheale* North American field isolates express a hemolysin-like protein Avian Dis 2010 54 3 994 1001 PMID: 20945779 10.1637/9070-091409-Reg.1 20945779

[B13] Ludwig W Euzéby J Whitman WB Krieg NR, Staley JT, Brown DR, Hedlund BP, Paster BJ, Ward NL, Ludwig W, Whitman WB Taxonomic outlines of the phyla *Bacteroidetes, Spirochaetes, Tenericutes (Mollicutes), Acidobacteria, Fibrobacteres, Fusobacteria, Dictyoglomi, Gemmatimonadetes, Lentisphaerae, Verrucomicrobia, Chlamydiae,* and *Planctomycetes* Bergey’s Manual of Systematic Bacteriology, Volume 4 2011 2 New York: Springer 21 2

[B14] Field D Garrity G Gray T Morrison N Selengut J Sterk P Tatusova T Thomson N Allen MJ Angiuoli SV Ashburner M Axelrod N Baldauf S Ballard S Boore J Cochrane G Cole J Dawyndt P De Vos P dePamphilis C Edwards R Faruque N Feldman R Gilbert J Gilna P Glöckner FO Goldstein P Guralnick R Haft D Hancock D The minimum information about a genome sequence (MIGS) specification Nat Biotechnol 2008 26 5 541 7 doi:10.1038/nbt1360 10.1038/nbt1360 18464787 PMC2409278

[B15] Garrity GM, Parker CT Nomenclature Abstract for "Bacteria". The NamesforLife Abstracts 2013 LLC: NamesforLife http://doi.org/10.1601/nm.419

[B16] Woese CR Kandler O Wheelis ML Towards a natural system of organisms: proposal for the domains *Archaea*, *Bacteria*, and *Eucarya* Proc Natl Acad Sci U S A 1990 87 4576 9 10.1073/pnas.87.12.4576 2112744 PMC54159

[B17] Garrity GM, Parker CT Nomenclature Abstract for "Bacteroidetes". The NamesforLife Abstracts 2013 LLC: NamesforLife http://doi.org/10.1601/nm.7927

[B18] Garrity GM Lilburn TG Cole JR Harrison SH Euzéby J Tindall BJ Taxonomic outline of the Bacteria and Archaea, Release 7.7 March 6, 2007; Part 1 - The “Archea”, Phyla “Crenarchaeota” and “Euryarchaeota” Taxonomic Outline 2007 551 73 doi:10.1601/TOBA7.7

[B19] Garrity GM, Parker CT Nomenclature Abstract for “Flavobacteriia”. The NamesforLife Abstracts 2014 LLC: NamesforLife http://doi.namesforlife.com/10.1601/nm.22978

[B20] Bernardet J-F Krieg NR, Staley JT, Brown DR, Hedlund BP, Paster BJ, Ward NL, Ludwig W, Whitman WB Class II. Flavobacteriia class. nov Bergey’s Manual of Systematic Bacteriology. 2nd ed. Volume 4 2011 New York: Springer 105

[B21] Garrity GM, Parker CT Nomenclature Abstract for Flavobacteriales. The NamesforLife Abstracts 2012 LLC: NamesforLife http://doi.org/10.1601/nm.8069

[B22] Bernardet J-F Krieg NR, Staley JT, Brown DR, Hedlund BP, Paster BJ, Ward NL, Ludwig W, Whitman WB Order I. *Flavobacteriales* ord. nov Bergey’s Manual of Systematic Bacteriology 2011 2 New York: Springer 105

[B23] Garrity GM, Parker CT Nomenclature Abstract for Flavobacteriaceae. The NamesforLife Abstracts 2014 LLC: NamesforLife http://doi.org/10.1601/nm.8070

[B24] Reichenbach H Holt JG Order 1. Cytophagales Leadbetter 1974, 99A Bergey’s Manual of Systematic Bacteriology. 1st ed. Volume 3 1989 Baltimore, MD: The Williams and Wilkins Co 2011 3

[B25] Bernardet JF Nakagawa Y Holmes B Proposed minimal standards for describing new taxa of the family Flavobacteriaceae, and emended description of the family Int J Syst Evol Microbiol 2002 52 1049 70 doi:10.1099/ijs. 0.02136-0 10.1099/ijs.0.02136-0 12054224

[B26] Garrity GM, Parker CT Nomenclature Abstract for Ornithobacterium. The NamesforLife Abstracts 2009 LLC: NamesforLife http://doi.org/10.1601/nm.8175

[B27] Vandamme P Segers P Vancanneyt M van Hove K Mutters R Hommez J Dewhirst F Paster B Kersters K Falsen E Devriese LA Bisgaard M Hinz K-H Mannheim W *Ornithobacterium rhinotracheale* gen. nov., sp. nov., isolated from the avian respiratory tract Int J Syst Bacteriol 1994 44 24 37 doi:10.1099/00207713-44-1-24 10.1099/00207713-44-1-24 8123560

[B28] BAuA) GFIfOSaH. 2010 Classification of prokaryotes (bacteria and archaea) into risk groups, technical rules for biological agents (TRBA) 466:159. Bundesanstalt für Arbeitsshutz und Arbeitsmedizin (BAuA http://www.baua.de/de/Startseite.html

[B29] Ashburner M Ball CA Blake JA Botstein D Butler H Cherry JM Davis AP Dolinski K Dwight SS Eppig JT Harris MA Hill DP Issel-Tarver L Kasarskis A Lewis S Matese JC Richardson JE Ringwald M Rubin GM Sherlock G Gene ontology: tool for the unification of biology Nat Biotechnol 2000 25 1 25 9 doi:10.1038/75556 10.1038/75556 PMC3037419 10802651

[B30] Chevreux B Wetter T Suhai S Genome sequence assembly using trace signals and additional sequence information Comp. Sci. Biol.: Proc. German Conference on Bioinformatics GCB ’99 (GCB) 1999 99 45 56 http://www.bioinfo.de/isb/gcb99/talks/chevreux/

[B31] Bonfield JK Whitwham A Gap5--editing the billion fragment sequence assemby Bioinformatics 2010 26 14 1699 703 doi:10.1093/bioinformatics/btq268 10.1093/bioinformatics/btq268 20513662 PMC2894512

[B32] Ronen R Boucher C Chitsaz H Pevzner P SEQuel: improving the accuracy of genome assemblies Bioinformatics 2012 28 12 i188 96 doi:10.1093/bioinformatics/bts219 10.1093/bioinformatics/bts219 22689760 PMC3371851

[B33] portal NWGSWgsp https://submit.ncbi.nlm.nih.gov/subs/wgs/

[B34] Petersen TN Brunak S von Heijne G Nielsen H SignalP 4.0: discriminating signal peptides from transmembrane regions Nat Methods 2011 8 10 785 6 doi:10.1038/nmeth.1701 10.1038/nmeth.1701 21959131

[B35] Krogh A Larson B von Heijne G Sonnhammer ELL Predicting transmembrane protein topology with a hidden Markov model: Application to complete genomes J Mol Biol 2001 305 3 567 80 doi:10.1006/jmbi.2000.4315 10.1006/jmbi.2000.4315 11152613

[B36] Grissa I Vergnaud G Pourcel C CRISPRFinder: a web tool to identify clustered regularly interspaced short palindromic repeats Nucleic Acids Res 2007 35 W52 7 doi:10.1093/nar/gkm360 10.1093/nar/gkm360 17537822 PMC1933234

[B37] Tamura K Peterson D Peterson N Stecher G Nei M Kumar S MEGA5: Molecular evolutionary genetics analysis using maximum likelihood, evolutionary distance, and maximum parsimony methods Mol Biol Evol 2011 28 10 2731 9 doi:10.1093/molbev/msr121 10.1093/molbev/msr121 21546353 PMC3203626

[B38] Zehr ES Bayles DO Boatwright WD Tabatabai LB Register KB **Complete genome sequence of**** *Ornithobacterium rhinotracheale* ****strain ORT-UMN 88. Stand** Genomic Sci 2014 9 16 doi:10.1186/1944-3277-9-16 10.1186/1944-3277-9-16 PMC4334632 25780507

